# Editorial: Pharmacoinformatics: new developments and challenges in drug design

**DOI:** 10.3389/fphar.2024.1462070

**Published:** 2024-07-30

**Authors:** David Ramírez, Rafael Peláez, Carlos F. Lagos, José L. Medina-Franco

**Affiliations:** ^1^ Departamento de Farmacología, Facultad de Ciencias Biológicas, Universidad de Concepción, Concepción, Chile; ^2^ Laboratorio de Química Orgánica y Farmacéutica, Departamento de Ciencias Farmacéuticas, Facultad de Farmacia, Universidad de Salamanca, Salamanca, Spain; ^3^ Instituto de Investigación Biomédica de Salamanca (IBSAL), Facultad de Farmacia, Universidad de Salamanca, Salamanca, Spain; ^4^ Centro de Investigación de Enfermedades Tropicales de la Universidad de Salamanca (CIETUS), Facultad de Farmacia, Universidad de Salamanca, Salamanca, Spain; ^5^ Chemical Biology and Drug Discovery Lab, Escuela de Química y Farmacia, Facultad de Medicina y Ciencia, Universidad San Sebastián, Campus Los Leones, Santiago, Chile; ^6^ Centro Ciencia and Vida, Fundación Ciencia and Vida, Huechuraba, Santiago, Chile; ^7^ DIFACQUIM Research Group, Department of Pharmacy, School of Chemistry, Universidad Nacional Autónoma de México, Mexico City, Mexico

**Keywords:** pharmacoinformatics, drug design, systems pharmacology, multi target directed ligand, pharmacology

Pharmacoinformatics represents a crucial nexus between computational sciences and pharmacology, aiming to leverage advanced data analysis and machine learning techniques to streamline drug discovery and development. The Research Topic, “Pharmacoinformatics: New Developments and Challenges in Drug Design,” brings together 10 original research articles contributed by more than 57 authors with over 18,000 views and downloads in all of the time until July 2024, that exemplify the transformative potential of computational approaches in modern pharmacology. The articles in this Research Topic underscore the diverse methodologies and applications of pharmacoinformatics, addressing a broad range of therapeutic areas and offering novel insights into drug design, efficacy, and mechanism of action.

One of the key contributions to this Research Topic is the study by She et al., titled “Deep learning-based multi-drug synergy prediction model for individually tailored anti-cancer therapies.” This paper presents a sophisticated deep learning model to predict synergistic multiple-drug combinations for cancer treatment. The model’s ability to tailor therapies to individual patients marks a significant step towards personalized medicine, demonstrating how machine learning can optimize therapeutic regimens and potentially improve clinical outcomes. The wide and appropriate use of artificial intelligence techniques such as machine learning techniques continues to revolutionize drug-disease association predictions, as demonstrated by Luo et al. in “Prediction of drug–disease associations based on reinforcement symmetric metric learning and graph convolution network.” The innovative use of reinforcement learning and graph convolution networks in this study highlights the evolving landscape of computational drug discovery, offering new avenues for identifying potential therapeutic applications of existing drugs.

Another compelling study, “Network pharmacology-based approach to explore the underlying mechanism of sinomenine on sepsis-induced myocardial injury in rats” by Sun et al., utilizes network pharmacology to unravel the mechanisms by which sinomenine exerts its protective effects against myocardial injury in sepsis. This research highlights the utility of network-based analyses in identifying key molecular interactions and pathways, providing a holistic understanding of drug action and facilitating the discovery of new therapeutic targets. The potential of network pharmacology is further exemplified by Li et al., where the authors studied the potential mechanisms of the anti-osteoporotic effects of the Achyranthes bidentata–Dipsacus asper herb pair. This research combines network pharmacology with experimental validation to decode the anti-osteoporotic mechanisms of a traditional herb pair, showcasing a robust approach to studying complex herbal formulations.

The investigation by Tran et al., “Identifying target organ location of Radix Achyranthis Bidentatae: a bioinformatics approach on active compounds and genes,” exemplifies the integration of bioinformatics in phytomedicine research. By identifying the target organs of herbal therapies this study not only advances our understanding of traditional medicinal herbs but also illustrates the broader applicability of pharmacoinformatics in natural product research.

In the field of antiviral drug discovery, Castillo-Campos et al. contribute a comprehensive computational analysis titled “Computational study of the binding orientation and affinity of noncovalent inhibitors of the papain-like protease (PLpro) from SARS-CoV-1 considering the protein flexibility by using molecular dynamics and cross-docking.” This work underscores the importance of accounting for protein flexibility in docking and molecular dynamics studies and provides valuable insights into the development of inhibitors against viral proteases, crucial for combating viral pathogens like SARS-CoV-1. In the same field, Castillo et al. discovered novel Mpro destabilizers with scope as broad-spectrum antivirals. They illustrate the innovative use of pharmacoinformatics for drug repurposing. By identifying Mpro destabilizers with potential broad-spectrum antiviral activity, this study contributes to the ongoing efforts to find effective treatments for viral infections.

Exploring the field of chemical libraries, Ginex et al. present two novel chemical libraries, MBC and ECBL as outstanding tools for drug discovery. Both libraries are designed to enhance the efficiency of drug discovery. The strategic design and diverse chemical space covered by these libraries are poised to accelerate the identification of promising drug candidates. The design of focused chemical libraries is further elaborated in Saldívar-González et al.’s work, “Design of a multi-target focused library for antidiabetic targets using a comprehensive set of chemical transformation rules.” By employing a multi-target approach, this study aims to address the multifactorial nature of diabetes, providing a valuable resource for the development of antidiabetic therapies.

Finally, in the synthesis and evaluation of novel compounds, Martín-Encinas et al.’s article, “Synthesis, biological and computational evaluation of novel cyanomethyl vinyl ether derivatives” stands out. This research integrates computational and experimental approaches to characterize new compounds, showcasing a comprehensive strategy for drug development.

Collectively, the contributions of the Research Topic, “Pharmacoinformatics: New Developments and Challenges in Drug Design,” highlight the dynamic and interdisciplinary nature of pharmacoinformatics. The integration of advanced computational methods with pharmacological research not only accelerates drug discovery but also enhances our understanding of complex biological systems. As we continue to navigate the challenges and opportunities in this field, the studies presented in this Research Topic open new avenues for future innovations in drug design and therapeutic interventions.

We hope that this Research Topic of articles inspires further research and collaboration, ultimately leading to the development of more effective and personalized therapeutic strategies.

